# Serum and urine lipidomic profiles identify biomarkers diagnostic for seropositive and seronegative rheumatoid arthritis

**DOI:** 10.3389/fimmu.2024.1410365

**Published:** 2024-05-03

**Authors:** Rong Li, Jung Hee Koh, Woo Jung Park, Yongsoo Choi, Wan-Uk Kim

**Affiliations:** ^1^ Natural Product Research Center, Korea Institute of Science and Technology (KIST), Gangneung, Republic of Korea; ^2^ Department of Marine Bio Food Science, Gangneung-Wonju National University, Gangneung, Republic of Korea; ^3^ Division of Rheumatology, Department of Internal Medicine, College of Medicine, The Catholic University of Korea, Seoul, Republic of Korea; ^4^ Center for Integrative Rheumatoid Transcriptomics and Dynamics, College of Medicine, The Catholic University of Korea, Seoul, Republic of Korea; ^5^ Division of National Product Applied Science, KIST School, Korea University of Science and Technology, Seoul, Republic of Korea

**Keywords:** rheumatoid arthritis, lipids, serum, urine, diagnosis

## Abstract

**Objective:**

Seronegative rheumatoid arthritis (RA) is defined as RA without circulating autoantibodies such as rheumatoid factor and anti-citrullinated protein antibodies; thus, early diagnosis of seronegative RA can be challenging. Here, we aimed to identify diagnostic biomarkers for seronegative RA by performing lipidomic analyses of sera and urine samples from patients with RA.

**Methods:**

We performed untargeted lipidomic analysis of sera and urine samples from 111 RA patients, 45 osteoarthritis (OA) patients, and 25 healthy controls (HC). These samples were divided into a discovery cohort (n = 97) and a validation cohort (n = 84). Serum samples from 20 patients with systemic lupus erythematosus (SLE) were also used for validation.

**Results:**

The serum lipidome profile of RA was distinguishable from that of OA and HC. We identified a panel of ten serum lipids and three urine lipids in the discovery cohort that showed the most significant differences. These were deemed potential lipid biomarker candidates for RA. The serum lipid panel was tested using a validation cohort; the results revealed an accuracy of 79%, a sensitivity of 71%, and a specificity of 86%. Both seropositive and seronegative RA patients were differentiated from patients with OA, SLE, and HC. Three urinary lipids showing differential expression between RA from HC were identified with an accuracy of 84%, but they failed to differentiate RA from OA. There were five lipid pathways that differed between seronegative and seropositive RA.

**Conclusion:**

Here, we identified a panel of ten serum lipids as potential biomarkers that can differentiate RA from OA and SLE, regardless of seropositivity. In addition, three urinary lipids had diagnostic utility for differentiating RA from HC.

## Introduction

Rheumatoid arthritis (RA) is a systemic inflammatory disease that affects mainly synovial joints. The autoimmune nature of the disease is supported by the presence of RA-associated autoantibodies such as anti-citrullinated peptide antibodies (ACPA) and rheumatoid factor (RF). Usually, a diagnosis of RA is based on clinical symptoms and serological positivity for these autoantibodies (1). ACPA and RF are present in approximately 70%–80% of patients with RA (2, 3). However, an estimated 20%–25% of cases of RA do not present with RF and ACPA in serum despite meeting the clinical classification criteria for RA (4); these patients are referred to as “seronegative”. Patients with seronegative RA experience delays in diagnosis and initiation of treatment with disease-modifying antirheumatic drugs (DMARDs) (5).

To facilitate diagnosis of seronegative RA, studies have explored potential biomarkers. For example, serum antibodies against disease-associated protein, including carbamylated proteins and against peptidyl-arginine deiminase type 4, have been investigated as potential markers; these biomarkers identified 23%–36% of patients with seronegative RA (6, 7). Recently, several proteomics or metabolomics studies identified biomarker candidates that can differentiate patients with seronegative RA from healthy controls (HC) (8, 9), patients with psoriatic arthritis (10), and patients with polymyalgia rheumatica (11).

Differential diagnosis of seronegative RA and OA of the hand also is challenging because inflammatory changes such as synovitis, tenosynovitis, effusion, and erosion are often observed in patients with OA (12); however, to the best of our knowledge, no study has attempted to identify markers that distinguishing seronegative RA from OA. The therapeutic strategies for the two diseases are different; early initiation of DMARD therapy is essential if patients with RA are to achieve remission and to limit joint damage (13), whereas DMARD therapy is not recommended for patients with OA (14).

Urine contains small hydrophilic molecules, including soluble lipids. Urine contains unwanted or excess compounds that are to be excreted from the body; therefore, it is a rich source of disease biomarkers (15). Furthermore, collection of large amounts of urine is noninvasive and convenient. Previously, we conducted a proteomic study showing that urinary soluble CD14 has diagnostic value as a strong predictor of RA disease activity (15); however, as far as we know, no study has conducted lipidomic analyses to identify biomarkers for RA.

Previously, we analyzed the integrative lipidome profile of patients with RA from the pre-clinical to sustained remission phase, and identified novel lipid biomarker candidates that predict development of RA in “seropositive” individuals with arthralgia (16). In the present study, we conducted a lipidome analysis to investigate lipid biomarker candidates that differentiate seropositive RA from seronegative RA and OA. We also conducted lipidome analysis of urine to assess its diagnostic utility. Subsequently, we validated biomarker candidates using an internal validation cohort. Furthermore, we investigated pathogenic differences between seropositive RA and seronegative RA by analyzing lipid metabolic pathways.

## Patients and methods

### Study participants

The study participants were enrolled in the Center for Integrative Rheumatoid Transcriptomics and Dynamics (CIRAD) cohort, a prospective cohort of RA patients at Seoul St. Mary’s Hospital that was started in 2015. All participants with RA fulfilled the 2010 American College of Rheumatology (ACR)/European League Against Rheumatism (EULAR) RA classification criteria (1). Blood samples were collected after an 8-h fast, after which sera were separated and stored at -20°C prior to subsequent analysis. Urine samples (midstream) were collected on the same day as blood samples. After centrifugation, the clarified supernatants were aliquoted and stored at −80°C until use. All patients had normal renal function and did not have clinically evident nephropathy.

The CIRAD cohort included patients with OA characterized by normal acute phase reactant levels without RF or ACPA. Serum and urine samples were collected at the same time. Healthy participants were recruited from the Wonju Severance Christian Hospital, Wonju, Gangwon-do; all were aged ≥ 20 years and had no known comorbidities or hand arthralgia.

Among the 136 RA patients enrolled from July 2019 to December 2020, 25 taking lipid-lowering agents were excluded from the analysis due to the potential impact of these drugs on the lipidome profile. Samples taken in 2019 (49 RA, 23 OA, 25 healthy individuals) were selected for the discovery cohort, and those taken in 2020 (62 RA and 22 OA) were selected as the internal validation cohort. Patients with RA in the validation cohort were classified as seropositive or seronegative to determine the clinical relevance of lipid candidates identified in the discovery cohort for differentiating seronegative RA from OA. Patients with seropositive RA were defined as patients who were positive for RF or ACPA, while patients with seronegative RA were defined as patients who were negative for RF and ACPA, although they met the 2010 ACR/EULAR RA classification criteria.

**Figure 1:**
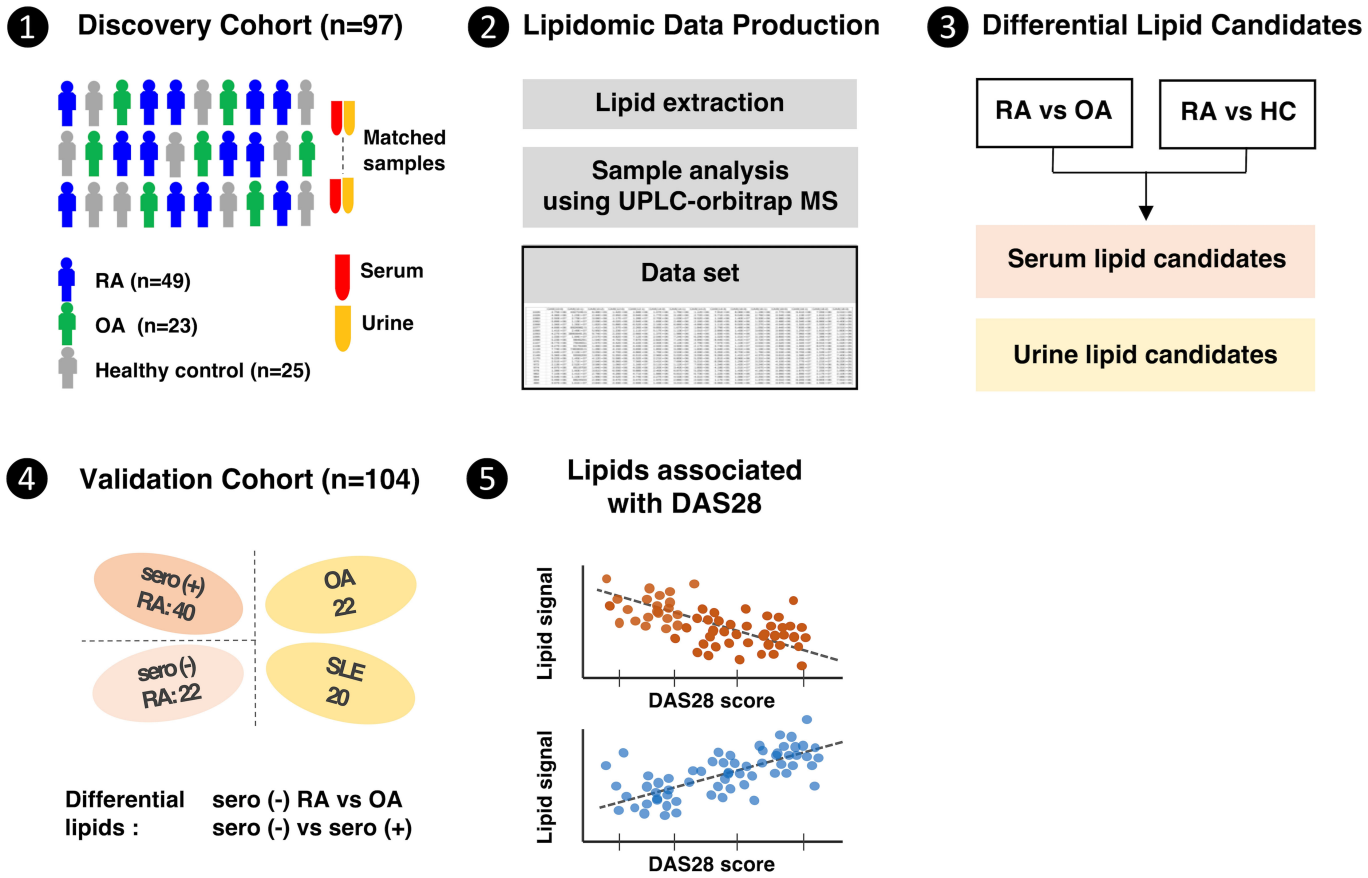
Clinical design and research flow of the comprehensive lipidome study in rheumatoid arthritis patients.

In addition, to assess whether the lipid biomarker candidates identified in the discovery cohort were specific for RA, anonymized serum samples from patients with systemic lupus erythematosus (SLE) were included in the validation cohort (**Figure 1**); although the SLE samples were initially obtained for use in an SLE-specific study, all patients consented to the use of these samples for other studies.

### Ethical approval

The study was approved by the institutional review board of Seoul St. Mary’s Hospital, the Catholic University of Korea (KC16SISI0632), and Wonju Severance Christian Hospital (19–008). All participants provided written informed consent in accordance with the Declaration of Helsinki. Informed consent was obtained before participation in the study.

### Preparation of lipid standards

Lipid standard mixtures were prepared at a stock concentration (5 μg/mL) and used as an internal standard comprising the following lipids: LPE 14:0, LPC 13:0, LPG 14:0, PA 10:0/10:0, Cer d18:1/12:0, PE 10:0/10:0, PG 10:0/10:0, Cer1P d18:1/12:0, PC 10:0/10:0, PS 10:0/10:0, SM d18:0/12:0, and TG 11:1/11:1/11:1. All lipid standards, with the exception of TG, were purchased from Avanti Polar Lipids (Alabaster, AL, USA). TG was acquired from Larodan Fine Chemicals AB (Malmö, Sweden).

### Lipid extraction from serum and urine samples

Lipids were extracted using the Folch method, with slight modifications (17). Briefly, a 60 μL aliquot of each serum or urine sample was mixed with 240 μL of ice-cold methanol, followed by centrifugation (16,000 rcf, 4°C, 10 min). The supernatant was then transferred into a 2.5 mL Eppendorf tube, to which 700 μL of chloroform:methanol mixture solvent (6:1, v/v) and 10 μL of lipid standard mixture were added, This was vortexed and incubated for 1 h at room temperature. Next, 180 μL of water was added to the tube, vortexed for 1 min, and subjected to centrifugation (16,000 rcf, 4°C, 10 min). The lower phase was collected using a glass pipette and transferred into a new tube for subsequent drying under nitrogen gas. The aqueous layer was re-extracted using the same procedure. Quality control samples were prepared by combining equal volumes of each serum or urine sample, followed by extraction in the same manner. All dried samples were stored at -20°C until analysis.

### Untargeted lipidomics using UPLC-ESI-MS/MS

The dried lipid extracts were reconstituted with 60 μL of isopropanol/methanol (1:1, v/v) and then separated on a Waters C18 column (Waters ACQUITY, 2.1 × 100 mm, 1.7 μm) connected to a Vanquish UPLC solvent delivery system (Thermo Scientific, San Jose, CA, USA). The binary solvent system included mobile phase A (ACN/H2O, 60:40, v/v) and mobile phase B (IPA/ACN, 90:10, v/v), both containing 10 mM ammonium formate and 0.1% formic acid. The flow rate for all separations was 0.33 mL/min, with the following elution gradient: 30% B for 1 min, followed by a linear increase from 30% to 98% B from 1 to 20 min. The UPLC column was re-equilibrated with 30% mobile phase B for 3 min between injections.

For MS data acquisition, a Q-Exactive Plus mass spectrometer (Thermo Scientific, San Jose, CA, USA) was operated in ESI-positive ion mode, performing a full MS mode from m/z 100-1000. To identify lipids, ten serum and urine samples underwent MS/MS data-dependent acquisition. The mass spectrometric parameters for both MS and MS/MS scans included a spray voltage at 3.5 kV, sheath gas at 40, auxiliary nitrogen pressures set at 10, capillary temperature at 320°C, and an S-lens radio frequency level at 50. Serum and urine samples were analyzed in two independent batches and in a random sequence.

To ensure consistency in large-scale lipidomic analysis, we divided the samples into two independent batches for discovery and validation cohorts. This approach mitigated inherent instrument fluctuations over an extended period (18). Additionally, we developed a randomized sequence analysis with serval sequence rows, each containing different types of RA, HC, and quality controls to prevent systematic errors that might occur when samples are analyzed in a specific order (19, 20). To further confirm the high quality and reliability of the data produced in each batch, all quality controls were ensured to occupy the same position in the multivariate analysis (19, 20).

### Data processing and statistical analysis

Lipid annotation was conducted by LipidSearch 4.0 (Thermo Scientific), based on the precursor ion and MS/MS fragmented ions with mass accuracy error < 5.0 ppm and 8.0 ppm, respectively. Raw data were imported into MZmine 2.53 for data preprocessing (21), and encompassed detection of targeted lipid features and peal alignment. Unreliable lipid signals were excluded if the relative standard deviation of the quality control samples exceeded 30%.

Statistical analysis was performed using MetaboAnalyst 5.0 (www.metaboanalyst.ca). Every lipid feature was normalized according to the median intensity of each sample, followed by log transformation and auto-scaling prior to multiple statistical analyses.

Lipids showing differential expression between two groups were identified by Orthogonal projections to latent structures-discriminate analysis (OPLS-DA), and a non-parametric Wilcoxon rank-sum t-test. The OPLS-DA model was validated using permutation tests (n=1000), and Q^2^ value was used to access overfitting. Potential lipid candidates were identified based on the following criteria: VIP > 1, P < 0.05, and FDR < 0.25. The results were visualized using R package prior to heatmap construction.

All lipids showing differential expression between RA and OA or HC were analyzed by Pearson’s correlation analysis to identify correlations with DAS28. Lipids with an absolute correlation coefficient (|r|) > 0.35 were considered to have a significant association with RA disease activity. Furthermore, receiver operating characteristic (ROC) curve analyses were performed to assess whether these lipids could differentiate between moderate-to-high disease activity and low disease activity or remission.

To prioritize lipid candidates, a multivariate exploratory ROC analysis was performed using MetaboAnalyst with classification and feature ranking of random forest. ROC curves based on prioritized lipids were generated using the pROC and randomForest package, and area under the curve (AUC) values were calculated using the random forest algorithm within the package.

To investigate whether lipid expression differed according to the serological status, the lipidome profiles of patients with seropositive RA and seronegative RA were compared. To avoid the possibility of bias caused by biological DMARDs, and to minimize the nonspecific effect of disease activity, patients treated with such drugs, as well as and those with high disease activity scores (i.e., DAS28 > 5.2) were excluded from analysis of the lipidome differences between seropositive and seronegative RA, respectively. Initially, lipids showing differential expression between the seronegative RA and seropositive RA groups were identified using a volcano plot prior to lipid ontology analysis. Lipid ontology analysis based on these differentially-expressed lipids was conducted using lipid ontology (LION) (22). Lipids showing significant differences, along with normalized peak intensities, were utilized to generate an enrichment table and a PCA heatmap. Any lipids that did not match entries in the LION database were excluded from further analysis.

## Results

### Baseline characteristics

In total, 111 patients with RA (mean age, 57 years; 84.7% female), 45 patients with OA (mean age, 56 years; 88.9% female), 25 HCs (mean age, 48 years; 100% female), and 20 patients with SLE (mean age, 42 years; 100% female) were included. The clinical parameters of the patients with RA in the discovery and validation cohorts were similar (**Table 1**); however, the HCs and patients with SLE were younger than patients with RA and OA.

**Table 1:** Clinical and demographic characteristics of the study populations.

	Discovery cohort				Validation cohort				
	RA (n = 49)	OA (n = 23)	HC (n = 25)	P-value†	RA (n =62)	OA (n = 22)	SLE (n = 20)	P-value†	P-value‡
Female, n (%)	40 (81.6)	21 (91.3)	25 (100)	0.090	54 (87.1)	18 (81.8)	20 (100)	0.162	0.597
Age, years	57.0 ± 12.3^*^	50.6 ± 10.5	47.5 ± 3.7^*^	<0.001	56.2 ± 12.1^*^	61.3 ± 9.6^*,§^	42.1 ± 7.9^§^	<0.001	0.658
BMI, kg/m^2^	22.9 ± 2.8	22.7 ± 2.9	23.9 ± 3.1	0.260	22.6 ± 2.5	22.9 ± 4.5	–	0.225	0.802
RA duration, years	7.9 ± 7.7		–	–	6.7 ± 8.5	–	–		0.176
RF-positive, n (%)	35 (71.4)		–	–	36 (58.1)	–	–		0.167
ACPA-positive, n (%)	38 (77.6)		–	–	40 (64.5)	–	–		0.149
ESR, mm/hr	19.5 ± 18.5		–	–	23.3 ± 18.9	–	–		0.247
CRP, mg/dL	0.9 ± 1.3		–	–	1.1 ± 1.5	–	–		0.646
DAS28	3.7 ± 1.5		–	–	3.6 ± 1.5	–	–		0.644
Glucocorticoids, n (%)	32 (65.3)		–	–	44 (71.0)	–	–		0.544
Methotrexate, n (%)	31 (63.3)		–	–	32 (51.6)	–	–		0.152
HCQ, n (%)	20 (40.8)		–	–	27 (43.5)	–	–		0.848
Sulfasalazine, n (%)	9 (18.4)		–	–	7 (11.3)	–	–		0.415
Leflunomide, n (%)	15 (30.6)		–	–	20 (32.3)	–	–		0.857
Biologics, n (%)	13 (26.5)		–	–	14 (22.6)	–	–		0.749

ACPA, anti-citrullinated peptide antibody; BMI, body mass index; CRP, C-reaction protein; ESR, erythrocyte sedimentation rate; DAS28, disease activity score in 28 joints; HCQ, hydroxychloroquine; RF, rheumatoid factor; OA, osteoarthritis; RA, rheumatoid arthritis, HC, healthy control.

^*,§^Significant difference between two values after post-hoc analysis (Dunn’s multiple comparison test).

^†^P value in each development and validation cohort calculated by one-way ANOVA.

^‡^P value between RA patients in development and validation cohort calculated by a t-test.

### Serum and urine lipidome profiles of RA, OA, and HC

**Figure 2:**
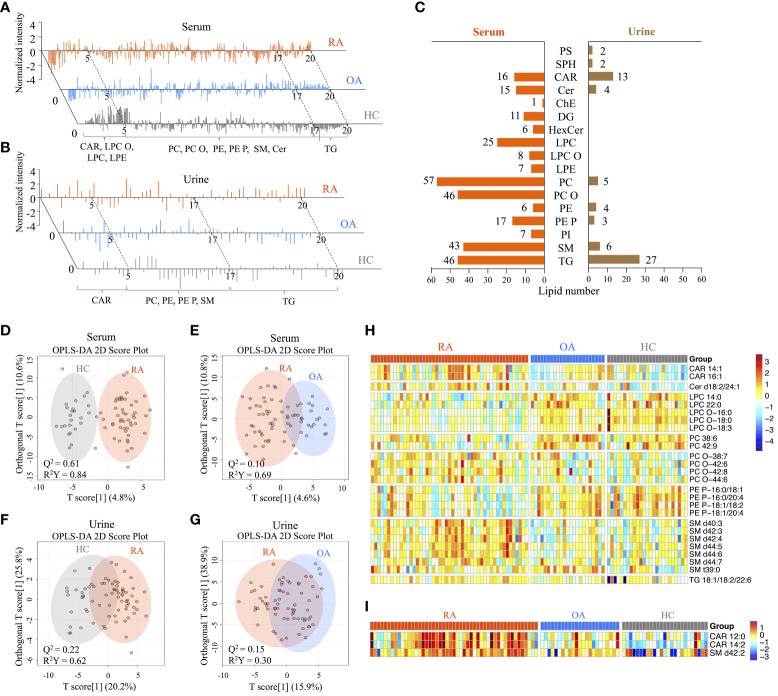
Comprehensive serum and urine lipidome profiles, and screening of differentially-expressed lipids between patients with rheumatoid arthritis (RA), healthy controls (HC), and patients with hand osteoarthritis (OA). **(A)** Representative LC-MS chromatograms showing the normalized intensity of lipids identified in serum and **(B)** urine lipid extracts from the RA, OA, and HC groups. **(C)** Distribution of lipid species in serum and urine samples. A total of 311 annotated serum and 61 urine lipids was categorized into 17 subclasses. **(D)** OPLS-DA 2D score plots based on the serum lipidome profiles between the RA and HC groups, and **(E)** between the RA and OA groups. **(F)** OPLS-DA 2D score plots based on the urine lipidome profiles between the RA and HC groups, and **(G)** between the RA and OA groups. The Q^2^ values indicate predictive ability derived from random permutation tests (n=1000). **(H)** Heatmap of normalized peak intensity shows significant differential expression of serum and **(I)** urine lipids in RA samples compared with OA and HC samples.

After data generation and processing, we identified a comprehensive array of lipids: 311 in serum and 66 in urine. Representative LC-MS chromatograms of serum and urine samples displayed marked differences in lipid profiles between RA and OA/HC (**Figures 2A, B**).

We classified these lipids into subclasses: 15 in serum and nine in urine (**Figure 2C**). Of the 15 subclasses in the serum lipidome, phosphatidylcholines (PC; 18%), ether-linked PCs (PC-Os; 15%), triacylglycerol (TG; 15%), and sphingomyelin (SM; 14%) were the most abundant. By contrast, TG (41%), carnitine (CAR; 20%), SM (9%), and PC (7.5%) were the most abundant in the urine lipidome.

The OPLS-DA 2D score plots for the serum lipidome demonstrated excellent separation of RA from HC, with a Q^2^ predictability of 0.61 (**Figure 2D**), as well as RA from OA, with a Q^2^ of 0.10 (**Figure 2E**). While the urine lipidome was less distinct than the serum lipidome, it still effectively differentiated RA from HC (Q^2^ = 0.22) and OA (Q^2^ = 0.15) (**Figures 2F, G**). Permutation tests (n = 1000) were performed to validate the OPLS-DA 2D models (**Supplementary Figure 1**). These tests confirmed the distinct serum and urine lipid profiles between RA patients and OA or HC, suggesting that there was no overfitting of the models when distinguishing RA from the two control groups.

Integrated analyses of OPLS-DA and Wilcoxon rank-sum tests between RA and OA or HC identified 26 lipids differentially expressed in serum and three in urine (**Table 2**). Among the serum lipids, ether-linked phosphatidylethanolamines (PE-Ps), PCs, lysophosphatidylcholines (LPCs), and ether-linked LPCs (LPC-Os) were significantly lower in patients with RA, while SMs, CARs, ceramide (Cer), and TG were higher, than in HC and OA (**Figure 2H**). The levels of these lipids, except SM t39:0, in OA patients were intermediate between RA patients and HC. SM t39:0 was significantly upregulated in patients with OA compared with HC, and LPC-O 18:0 was downregulated in OA (**Table 2**). Urine CAR 12:0, CAR 14:2, and SM d42:2 were higher in patients with RA than in OA and HC (**Figure 2I**).

**Table 2:** Differential lipid profiles in serum and urine from RA patients.

Lipids	RA/HC				RA/OA				OA/HC			
	VIP	Log_2_	P-value	FDR	VIP	Log_2_	P-value	FDR	VIP	Log_2_	P-value	FDR
Serum												
CAR 14:1	1.59	0.70	1.09 e^-2^	8.04 e^-2^	1.37	0.73	1.96 e^-2^	1.71 e^-1^	0.61	-0.04	4.49 e^-1^	9.12 e^-1^
CAR 16:1	2.16	0.71	5.44 e^-4^	1.06 e^-2^	1.64	0.52	8.72 e^-3^	1.18 e^-1^	1.00	0.19	2.77 e^-1^	8.35 e^-1^
Cer d18:2/24:1	2.68	0.42	8.73 e^-6^	3.39 e^-4^	2.27	0.26	2.76 e^-3^	6.61 e^-2^	1.38	0.16	1.44 e^-1^	6.88 e^-1^
LPC 14:0	1.84	-0.39	5.74 e^-3^	5.76 e^-2^	1.75	-0.33	9.39 e^-3^	1.21 e^-1^	0.12	-0.05	7.90 e^-1^	9.84 e^-1^
LPC 22:0	1.50	-0.27	1.67 e^-2^	1.04 e^-1^	2.07	-0.30	1.01 e^-2^	1.21 e^-1^	0.29	0.03	8.06 e^-1^	9.84 e^-1^
LPC O-16:0	2.41	-0.63	1.60 e^-6^	9.96 e^-5^	1.84	-0.26	1.88 e^-3^	6.49 e^-2^	1.45	-0.37	6.91 e^-2^	5.97 e^-1^
LPC O-18:0	2.76	-0.76	1.15 e^-8^	3.58 e^-6^	1.45	-0.27	3.49 e^-2^	2.26 e^-1^	2.18	-0.49	3.24 e^-3*^	1.44 e^-1^
LPC O-18:3	2.06	-0.70	1.46 e^-4^	3.79 e^-3^	2.05	-0.35	4.71 e^-3^	8.62 e^-2^	0.82	-0.35	2.42 e^-1^	8.25 e^-1^
PC 38:6	1.01	-0.20	3.29 e^-2^	1.46 e^-1^	1.82	-0.36	6.45 e^-3^	1.00 e^-1^	1.10	0.16	3.35 e^-1^	8.47 e^-1^
PC 42:9	1.65	-0.28	1.86 e^-2^	1.08 e^-1^	1.79	-0.24	3.35 e^-2^	2.22 e^-1^	0.11	-0.04	9.84 e^-1^	9.93 e^-1^
PC O-38:7	1.80	0.24	1.20 e^-2^	8.13 e^-2^	1.17	0.19	4.44 e^-2^	2.50 e^-1^	0.08	0.05	9.51 e^-1^	9.93 e^-1^
PC O-42:6	1.79	0.26	5.14 e^-3^	5.51 e^-2^	1.51	0.22	3.28 e^-2^	2.22 e^-1^	0.11	0.04	6.09 e^-1^	9.65 e^-1^
PC O-42:8	1.96	0.36	4.26 e^-3^	4.90 e^-2^	1.35	0.23	1.49 e^-2^	1.41 e^-1^	0.11	0.13	7.90 e^-1^	9.84 e^-1^
PC O-44:6	1.79	0.26	1.16 e^-2^	8.04 e^-2^	1.72	0.22	1.77 e^-2^	1.62 e^-1^	0.23	0.04	6.23 e^-1^	9.65 e^-1^
PE P-16:0/18:1	1.10	-0.28	4.50 e^-2^	1.77 e^-1^	1.35	-0.28	2.47 e^-2^	1.92 e^-1^	0.00	-0.01	8.38 e^-1^	9.84 e^-1^
PE P-16:0/20:4	1.64	-0.56	3.40 e^-4^	8.13 e^-3^	1.15	-0.25	2.99 e^-2^	2.11 e^-1^	1.60	-0.31	5.18 e^-2^	5.92 e^-1^
PE P-18:1/18:2	1.02	-0.51	2.51 e^-2^	1.22 e^-1^	1.96	-0.46	6.51 e^-4^	4.25 e^-2^	0.24	-0.05	6.68 e^-1^	9.65 e^-1^
PE P-18:1/20:4	1.00	-0.39	2.40 e^-2^	1.22 e^-1^	1.25	-0.33	2.10 e^-2^	1.72 e^-1^	0.21	-0.06	8.22 e^-1^	9.84 e^-1^
SM d40:3	1.88	0.30	3.51 e^-3^	4.37 e^-2^	2.08	0.34	6.83 e^-4^	4.25 e^-2^	0.47	-0.04	4.99 e^-1^	9.56 e^-1^
SM d42:3	2.17	0.26	9.35 e^-4^	1.71 e^-2^	2.18	0.24	1.72 e^-3^	6.49 e^-2^	0.17	0.02	8.70 e^-1^	9.84 e^-1^
SM d42:4	1.40	0.27	1.16 e^-2^	8.04 e^-2^	2.71	0.46	1.46 e^-5^	4.54 e^-3^	1.68	-0.19	5.99 e^-2^	5.92 e^-1^
SM d44:5	1.43	0.15	4.62 e^-2^	1.80 e^-1^	2.20	0.21	1.72 e^-3^	6.49 e^-2^	0.68	-0.05	2.96 e^-1^	8.35 e^-1^
SM d44:6	1.54	0.24	7.14 e^-3^	6.94 e^-2^	2.58	0.36	4.71 e^-5^	7.33 e^-3^	1.05	-0.13	1.50 e^-1^	6.88 e^-1^
SM d44:7	1.14	0.21	3.29 e^-2^	1.46 e^-1^	1.42	0.20	2.03 e^-2^	1.71 e^-1^	0.05	0.00	9.02 e^-1^	9.92 e^-1^
SM t39:0	1.49	0.29	1.90 e^-2^	1.08 e^-1^	1.56	-0.21	4.19 e^-2^	2.50 e^-1^	3.10	0.49	2.58 e^-4*^	2.68 e^-2^
TG 18:1/18:2/22:6	1.37	0.36	2.59 e^-2^	1.22 e^-1^	1.37	0.38	2.90 e^-2^	2.11 e^-1^	0.93	-0.02	9.35 e^-1^	9.92 e^-1^
Urine												
CAR 12:0	1.18	1.07	3.05 e^-5^	6.70 e^-4^	1.21	0.66	7.51 e^-3^	9.91 e^-2^	1.16	0.42	1.13 e^-1^	2.01 e^-1^
CAR 14:2	1.44	1.64	3.01 e^-8^	1.99 e^-6^	1.50	1.08	3.97 e^-4^	2.62 e^-2^	1.62	0.57	4.31 e^-3^	2.84 e^-2^
SM d42:2	1.32	0.72	8.23 e^-3^	1.51 e^-2^	1.27	0.90	6.70 e^-3^	9.91 e^-2^	0.87	-0.18	6.23 e^-1^	7.22 e^-1^

This table lists 26 differentially-expressed serum lipid candidates and three urine lipid candidates identified in RA patients but not in HC and OA patients.

Serum and urine lipids with a VIP >1, a p value < 0.05, and an FDR < 0.25 were considered to be biomarker candidates that discriminate RA from HC and OA. The p and FDR values were calculated using a non-parametric Wilcoxon rank-sum test.

CAR, acylcarnitine; Cer, ceramide; FDR, false discovery rate; LPC, lysophosphatidylcholine; LPC-O, ether-linked lysophosphatidylcholine; PC, phosphatidylcholine; PC O, ether-linked phosphatidylcholine; PS, phosphatidylserine; PE P, ether-linked phosphatidylethanolamine; SM, sphingomyelin; TG, triacylglycerol; VIP, variable importance in projection;

*Significantly different between patients with OA and HC.

### Diagnostic potential of lipid biomarkers for distinguishing RA

**Figure 3:**
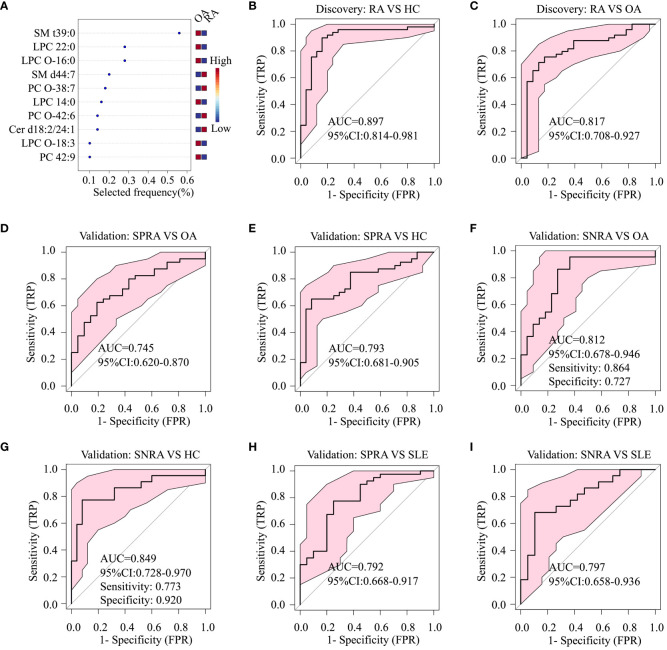
Diagnostic performance of serum lipid biomarker candidates based on ROC analysis. **(A)** Top ten serum lipids predicted by a random forest multivariate algorithm to distinguish between the RA and OA groups. Random forest analysis was performed using the 26 differentially-expressed serum lipids, including SM t39:0, LPC 22:0, SM d44:7, and PC O-38.7, listed in **Table 1**. **(B)** ROC analysis to identify differences between RA and HC, and **(C)** between RA and OA, in the discovery cohort. **(D)** ROC analysis to identify differences between seropositive RA (SPRA) and OA, **(E)** between SPRA and HC, **(F)** between seronegative RA (SNRA) and OA, and **(G)** between SNRA and HC in the validation cohort. **(H)** ROC analysis to identify differences between SPRA and SLE, and **(I)** between SNRA and SLE in the validation cohort.

Multivariate analyses using ROC AUC curves were conducted to pinpoint serum lipid biomarkers with the highest potential for distinguishing between RA and OA (**Supplementary Figure 2**). Feature ranking, conducted through the random forest algorithm, identified ten primary lipids as significant markers: PC 42:9, PC-O (38:7, 42:6), LPC (14:0, 22:0), LPC-O (16:0, 18:3), SM (d44:7, t39:0), and Cer d18:2/24:1 (**Figure 3A**). These biomarker candidates effectively differentiated RA patients from HC (AUC=0.897; 95% confidence interval (CI), 0.814–0.981) and OA (AUC=0.817; 95% CI, 0.708–0.927) in the discovery cohort (**Figures 3B, C**). Next, we evaluated these 10 in the validation cohort (**Figures 3D–I**).

**Figure 4:**
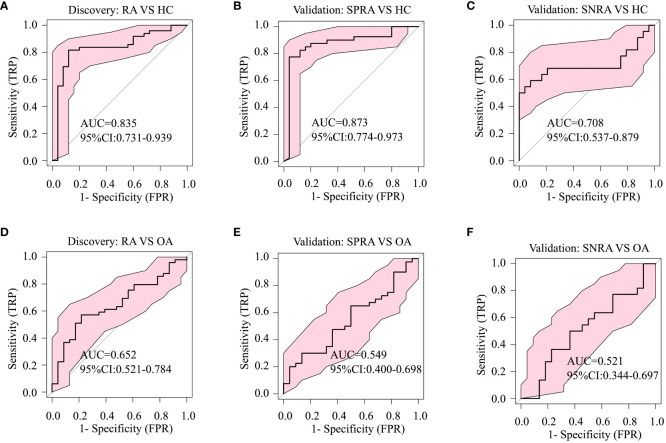
Diagnostic performance of urine biomarker candidates based on ROC analysis. The three differentially-expressed urine lipids CAR 12:0, CAR 14:2, and SM d42:2 listed in **Table 1** were used for ROC analysis of group classifications. **(A)** ROC analysis to identify differences between RA and HC in the discovery cohort, **(B)** between seropositive RA (SPRA) and HC, and **(C)** between seronegative RA (SNRA) and HC in the validation cohort. **(D)** ROC analysis to identify differences between RA and OA in the validation cohort, **(E)** between seronegative RA (SNRA) and HC and **(F)** between SNRA and OA in the validation cohort.

To ascertain whether these serum lipid biomarker candidates could be also used to discriminate patients with seronegative RA from those with OA or HC, we conducted a comparative analysis (**Figures 3F, G**). The serum lipid biomarker candidates demonstrated an AUC of 0.849, with a test sensitivity of 77% and specificity of 92%, between seronegative RA and HC, and an AUC of 0.812 (sensitivity, 86%; specificity, 73%) between seronegative RA and OA. Additionally, these markers were effective at differentiating RA from SLE, with an AUC of 0.797 (**Figures 3H, I**). Three upregulated urine lipids showed excellent performance for discriminating RA from HC (**Figures 4A–C**); however, they were suboptimal for discriminating RA from OA (**Figures 4D–F**).

### Association of lipidome profiles with disease activity

**Figure 5:**
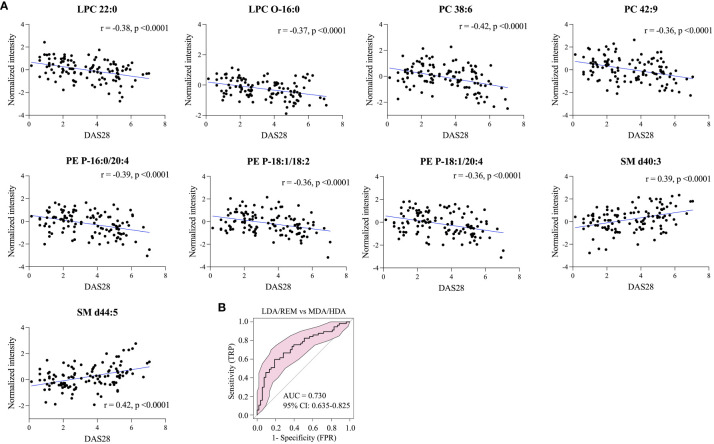
Lipids correlated with RA disease activity. **(A)** Nine representative lipids reflecting disease activity, and **(B)** model evaluation of RA groups by ROC analysis of moderate-to high disease activity (MDA/HDA) versus low disease activity or remission (LDA/REM).

Pearson correlation analysis of 26 differentially-expressed lipids from 113 RA patients was conducted to examine correlations with the DAS28 scores. Among these, nine lipids showed a significant correlation with DAS28 (|r| > 0.35). LPC 22:0, LPC-O 16:0, PC (38:6 and 42:9), and PE-P (16:0/20:4, 18:1/18:2, and 18:1/20:4) showed a negative correlation, whereas SM (d40:3 and d44:5) showed a positive correlation (**Figure 5A**). ROC curves based on these nine lipids differentiated patients with low disease activity or remission from patients with moderate-to-high disease activity (AUC, 0.730; 95% CI, 0.635−0.825) (**Figure 5B**).

### Comparison of the serum and urine lipid profiles of patients with seropositive RA with those of patients with seronegative RA

Next, we focused on elucidating lipidomic disparities between RA patients with seropositive or seronegative disease. There was no difference in the baseline characteristics, including age, sex, and disease activity, between the groups (**Supplementary Table 1**).

**Figure 6:**
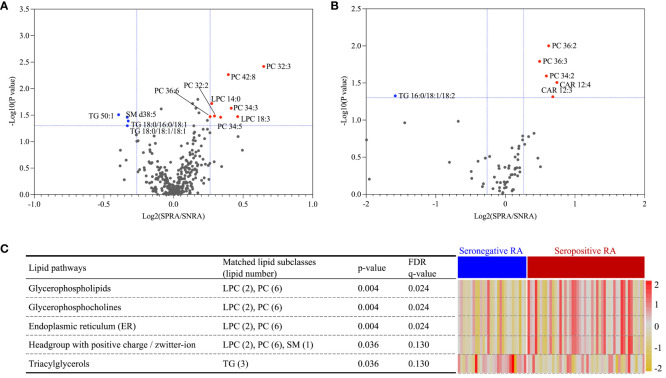
Lipid ontology enrichment analysis based on differential expression of serum lipids separating seropositive and seronegative RA. A volcano plot identified 12 and six lipids differentially expressed between seropositive and seronegative RA in serum **(A)** and urine **(B)**, respectively. Expression of five serum lipid-related pathways, shown as a heatmap **(C)**; data were obtained from lipid ontology enrichment analysis of all 12 serum lipids identified in the seropositive and seronegative RA groups.

Volcano plot analysis identified 12 serum and six urine lipids showing significant differences between patients with seronegative and seropositive RA (criteria of p<0.05 and FC>1.2 or < 0.8). In serum, eight lipids (including PCs and LPCs) were upregulated, and four (including TG and SM) were downregulated, in patients with seropositive RA (**Figure 6A**). In urine, three PCs (34:2, 36:2, 36:3) and two CARs (12:3, 12:4) were upregulated, and TG (16:0/18:1/18:2) was downregulated, in patients with seropositive RA (**Figure 6B**).

LION enrichment analysis of these 12 serum lipids (**Figure 6C**) revealed that among five significantly enriched pathways, the TG lipid pathway was the only upregulated pathway in patients with seronegative RA. Four lipid pathways, glycerophospholipids, glycerophosphocholines, endoplasmic reticulum, and a headgroup with a positive charge/Zwitterion, were upregulated in patients with seropositive RA.

## Discussion

There are far fewer studies of seronegative RA than seropositive RA; one of the reasons for this is the fear of misclassification (23). Here, we investigated the lipidome in patients with RA, OA, and HC, and identified candidate lipid biomarkers that can aid diagnosis of seronegative RA. Additionally, urinary lipids exhibited diagnostic potential for distinguishing RA from HC, suggesting a promising non-invasive diagnostic approach.

The downregulated lipid subclasses identified herein, which included LPCs, LPC-Os, and PC, in patients with RA were consistent with those identified in our previous study (16), which showed that the CAR and SM subclasses were elevated in inflammatory synovial fluid from RA patients (16). In line with these previous results, we found notable increases in CAR and SM subclasses in sera from RA patients. CAR is a biomarker associated with mitochondrial function (24). Mitochondrial dysfunction in RA fibroblast-like synoviocytes activates downstream proinflammatory pathways (25). Moreover, a recent study showed that disruption of SM is associated with progression and activity of RA (26). The results presented herein suggest that increased expression of CAR and SM in serum reflects their involvement in RA pathogenesis within the joint microenvironment.

We identified and validated three urine lipid biomarker candidates that distinguish patients with RA from HC. These lipids were CAR 12:0, CAR 14:2, and SM d42:2, and all were upregulated in patients with RA. Although serum and urine samples were collected on the same day and under the same conditions, lipids differentially expressed between RA and OA or HC were not the same in both samples. This suggests that many lipids present in serum are not excreted in urine. The CAR and SM subclasses were abundant in both urine and serum from RA patients; thus, we assume that CAR and SM are so abundant in RA patients that a proportion is secreted in the urine. The number of urine lipids was relatively low, reflecting the lower total lipid concentration in urine samples compared to plasma samples (27).

Interestingly, LPC-O 18:0 and SM t39:0 showed significant differential expression not only between patients with RA and OA, but also between patients with OA and HC. Overall, lipids differentially expressed between patients with RA and OA showed greater disparity than those differentially expressed between patients with OA and HC. OA is a prototype degenerative condition, and yet, it is accompanied by synovial inflammation, which itself is associated with radiographic progression (28). CRP levels in OA patients included in this study were all within the normal range; however, lipid profiles, which may reflect inflammation, showed different expression levels in OA patients and HC, although these differences were not as pronounced as RA. Moreover, the differences in lipid profiles between OA and HC suggest the potential for detecting OA-related inflammation using lipid biomarkers.

The levels of nine serum lipids correlated with RA disease activity: LPC, LPC-O, PC, and PE-P correlated negatively with DAS28, whereas SM correlated positively. Although this result did not completely reproduce our previous research findings (16), it showed a consistent tendency between lipid subclasses and RA disease activity.

The lipid profiles of patients with seronegative and seropositive RA were similar, although some lipids showed differential expression. This result suggests that lipid metabolism varies according to the presence of serological markers such as RF or ACPA, not just with the degree of inflammation. The development of seronegative RA seems to be related to lower genetic susceptibility and, more critically, to environmental factors (29). Moreover, monocytes and macrophages are predominant in seronegative synovitis, whereas lymphoplasmacytic infiltrates are more pronounced in seropositive synovitis (29). The different lipid pathways may reflect a different pathogenesis underlying seronegative RA. TGs were the only upregulated lipid in seronegative RA compared with seropositive RA. TGs are used for energy storage in adipose tissue (30). Dyslipidemia, which is considered to be an environmental factor, increases articular damage by activating the synovial mononuclear phagocyte system (31). Moreover, dysregulated adipose tissue secretes adipokines that promote systemic inflammation (32). Thus, upregulation of TGs in seronegative RA may be associated with histopathologic characteristics.

Among distinct lipid pathways observed between seropositive and seronegative RA, upregulation of the ER lipid pathway in seropositive RA suggests a possible link between lipids and the serological status of RA. ER is the intracellular organelle responsible for lipid synthesis and protein folding (33). GRP78/BiP, a representative ER-resident chaperone, is overexpressed in the synovial lining and sub-lining layer (34). As an extracellular protein, GRP78/BiP acts as putative autoantigen in RA (35). The citrullinated BiP protein is mostly expressed on the surface of synovium and provides a target for ACPA to activate the NF-ĸB proinflammatory pathway and TNF secretion (33–36). Thus, upregulation of ER-related lipids in patients with seropositive RA compared with seronegative RA may reflect this mode of pathogenesis.

This study has some limitations. First, samples were obtained from patients attending a real-world outpatient clinic; thus, most patients were receiving treatment. Lipid profiles are affected by medications, although we found no statistical difference in medication use between seronegative and positive RA patients. Therefore, we cannot exclude potential effects of medications. In our previous study, we found that the lipid profiles of each group (classified according to disease activity), was significantly different, even though they were taking similar medications (16). Therefore, we believe that DMARDs, glucocorticoids, and NSAIDs have minor effects on lipid profiles. Second, we recognized the significant heterogeneity of lipid constituents among individuals within the same group. It can affect the power of accuracy of lipid biomarker candidates in the validation cohort. As insoluble lipids are transported in association with proteins, protein-lipid connectivity networks provide further insights into lipid and protein constituents unique to metabolic characteristics (37, 38). There is a lack of prospective proteomics and lipidomics studies in RA. Thus, integrated lipidomics and proteomics can enhance the overall sensitivity and diagnostic accuracy of biomarker candidates by capturing a broader spectrum of biomolecular changes associated with seronegative RA.

This study has several strengths. First, the most significant finding of this study is identification of serum lipid biomarker candidates that can distinguish seronegative RA from OA. Second, to the best of our knowledge, this is the first parallel investigation of the urine and serum lipid lipidomes in patients with RA, enabling investigation of the relationship between the two different types of biofluid. Urine is the most noninvasive and readily obtainable biofluid for diagnosis; therefore, discovery of three lipid biomarker candidates for diagnosing RA is of some importance. Third, the lipid biomarker candidates were verified in the validation cohort comprising patients with seropositive and seronegative RA or OA, with SLE as an autoimmune disease control. If the lipid biomarker candidates are further validated in an external validation cohort, they can be applied in the real-world clinics.

In conclusion, we identified a serum lipidome signature that can distinguish patients with RA from HC and those with OA, irrespective of the serological status of RA. Some serum lipid profiles and pathways differed between patients with seropositive and seronegative RA, suggesting variations in lipid metabolism according to serological status. Three urinary lipids had diagnostic value for differentiating RA from HC.

## Data availability statement

The original contributions presented in the study are included in the article/**Supplementary Material**. Further inquiries can be directed to the corresponding authors.

## Ethics statement

The studies involving humans were approved by Seoul St.Mary’s hospital, the Catholic University of Korea and Wonju Severance Christian Hospital. The studies were conducted in accordance with the local legislation and institutional requirements. The participants provided their written informed consent to participate in this study.

## Author contributions

RL: Formal Analysis, Investigation, Methodology, Software, Visualization, Writing – original draft, Writing – review & editing. JK: Conceptualization, Data curation, Formal Analysis, Funding acquisition, Investigation, Project administration, Resources, Supervision, Validation, Writing – original draft, Writing – review & editing. WP: Conceptualization, Methodology, Software, Supervision, Visualization, Writing – original draft, Writing – review & editing. YC: Conceptualization, Data curation, Formal Analysis, Funding acquisition, Investigation, Methodology, Resources, Supervision, Validation, Visualization, Writing – original draft, Writing – review & editing. W-UK: Conceptualization, Formal Analysis, Funding acquisition, Investigation, Methodology, Project administration, Resources, Supervision, Validation, Visualization, Writing – original draft, Writing – review & editing.
