# Supplementary material for: Serum and urine lipidomic profiles identify biomarkers diagnostic for seropositive and seronegative rheumatoid arthritis
Source: Front Immunol. 2024 May 3;15:1410365. doi: 10.3389/fimmu.2024.1410365 (PMC11099275; doi:10.3389/fimmu.2024.1410365)
Supplement: Supplementary file 1 [file DataSheet_1.docx]

**Supplemental Information**

**Serum and urine lipidomic profiles identify biomarkers diagnostic**

**for seropositive and seronegative RA**

Rong Li^1,2*^, Jung Hee Koh^3,4*^, Woo Jung Park^2^, Yongsoo Choi^1,5†^, Wan-Uk Kim^3,4†^

^1^Natural Product Research Center, Korean Institute of Science and Technology (KIST), Gangneung, 25451, Korea

^2^ Department of Marine Food Science and Technology, Gangneung-Wonju National University, Gangneung, Korea, 25457, Korea

^3^ Division of Rheumatology, Department of Internal Medicine, the Catholic University of Korea, Seoul 06591, Korea

^4^ Center for Integrative Rheumatoid Transcriptomics and Dynamics, the Catholic University of Korea, Seoul 06591, Korea

^5^ Division of National Product Applied Science, KIST School, Korea University of Science and Technology, Seoul, 02792, Korea

^*,†^ These authors contributed equally to this work.

Correspondence and reprint requests to:

Professor Wan-Uk Kim, M.D., Ph.D.

Division of Rheumatology, Department of Internal Medicine, Seoul St. Mary’s Hospital, Center for Integrative Rheumatoid Transcriptomics and Dynamics, College of Medicine, The Catholic University of Korea, 222 Banpo-daero, Seocho-gu, Seoul 08826, Republic of Korea. E-mail: wan725@catholic.ac.kr, Tel.: +82-2-2258-7530, Fax: +82-2-2258-7526

Or

Professor Yongsoo Choi

Natural Product Research Center, Korean Institute of Science and Technology, 679, Saimdang-ro, Gangneung, Gangwon—do, 25451, Republic of Korea.

E-mail:Yongsoo.choi@kist.re.kr, Tel.: +82-33-650-3505, Fax: +82-33-650-3629


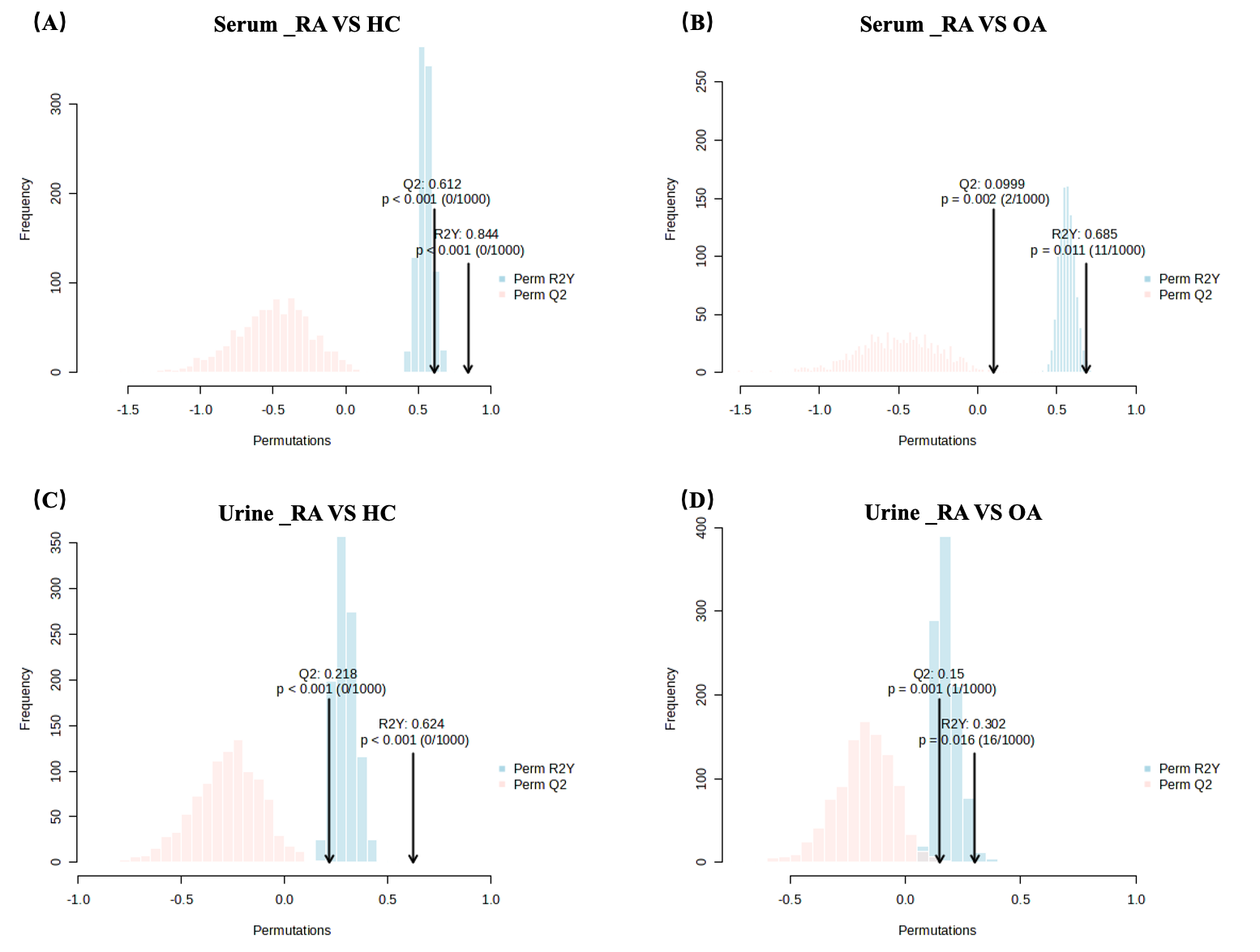


Supplemental Figure S1. OPLS-DA model validation by the permutation test based on 1000 permutations of lipids obtained by LC-MS analysis of serum samples from two different comparisons: (A) RA versus HC, (B) RA versus OA; and urine sample from two different comparisons: (C) RA versus HC, (D) RA versus OA. R^2^ value represents the goodness of fit and a Q^2^ value represents the predictive ability of the model.


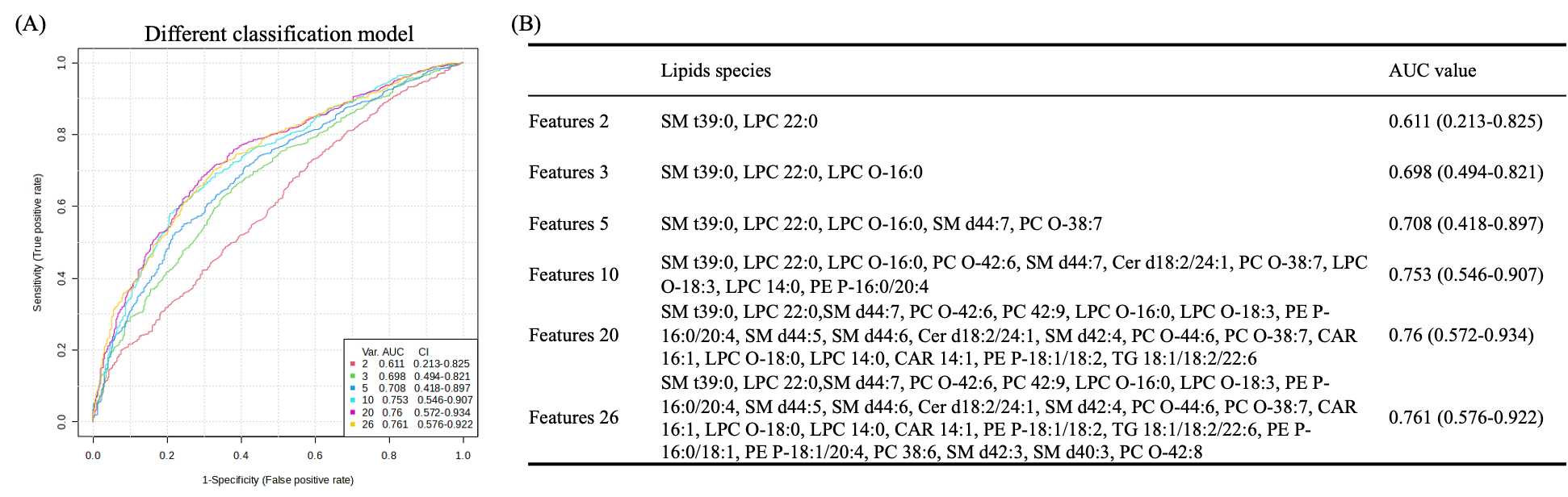


Supplemental Figure S2. Biomarker prediction by Multivariate ROC curve based exploratory analysis to find the most significant lipids that contribute differentiation of RA from OA. The ROC curves were generated based on an algorithm Monte-Carlo cross validation (MCCV) through balanced subsampling coupled with Random Forests for classification method and random forest built-in was selected as feature ranking method. (A) Overview of all ROC curves generated by MetaboAnalyst 5.0 from 6 different biomarker models using different number of lipid features (2, 3, 5, 10, 20 and 26) with corresponding AUC value and confidence interval; (B) 6 different models based on different number and species of lipid candidates including the individual lipids.

**Supplemental Table S1. The clinical information of patients with seropositive and seronegative RA**

|  | **Seropositive RA**  **(n = 48)** | **Seronegative RA**  **(n = 28)** | **P-value** |
| --- | --- | --- | --- |
| Female, n (%) | 47 (90.4) | 22 (78.6) | 0.532 |
| Age, years | 54.6 ±14.1 | 56.1 ± 13.8 | 0.655 |
| BMI, kg/m^2^ | 22.1 ± 3.4 | 22.4 ± 2.2 | 0.935 |
| Duration, years | 5.0 ± 5.4 | 5.2 ± 5.4 | 0.718 |
| ACPA-positive, n (%) | 41 (85.0) | - |  |
| RF-positive, n (%) | 46 (95.8) | - |  |
| DAS28 | 3.4 ± 1.3 | 2.8 ±1.4 | 0.053 |
| ESR, mm/hr | 17.8 ± 15.3 | 14.7 ± 15.6 | 0.186 |
| CRP, mg/dL | 0.7 ± 1.0 | 1.0 ± 1.8 | 0.583 |
| Medications |  |  |  |
| Methotrexate, n (%) | 29 (60.4) | 16 (57.1) | 0.813 |
| Leflunomide, n (%) | 18 (37.5) | 14 (50.0) | 0.340 |
| Sulfasalazine, n (%) | 5 (10.4) | 8 (28.6) | 0.059 |
| Hydroxychloroquine, n (%) | 28 (58.3) | 13 (46.4) | 0.348 |
| Glucocorticoids, n (%) | 36 (75.0) | 23 (82.1) | 0.575 |

ACPA: anti-citrullinated peptide antibody, BMI: body mass index, CRP: C-reaction protein, DAS28: disease activity score in 28 joints, ESR: erythrocyte sedimentation rate, NSAID: non-steroidal anti-inflammatory drugs, RF: rheumatoid factor.

P value was calculated by Mann-Whitney U test or Chi-square test, as appropriate.
